# A team level participatory approach aimed at improving sustainable employability of long-term care workers: a study protocol of a randomised controlled trial

**DOI:** 10.1186/s12889-022-13312-8

**Published:** 2022-05-16

**Authors:** Ceciel H. Heijkants, Madelon L. M. van Hooff, Sabine A. E. Geurts, Cécile R. L. Boot

**Affiliations:** Radboud University, Behavioural Science Institute, Thomas van Aquinostraat 4, Nijmegen, 6525GD The Netherlands

**Keywords:** Participatory workplace intervention, Self-managing teams, Study protocol, Randomised controlled trial, Need for recovery, Basic psychological needs, Autonomy, Relatedness, Competence

## Abstract

**Background:**

Staff currently working in long-term care experience several difficulties. Shortage of staff and poor working conditions are amongst the most prominent, which pose a threat to staff’s sustainable employability. To improve their sustainable employability it is important to create working conditions that fulfil workers’ basic psychological need for autonomy, relatedness and competence in line with Self-Determination Theory. Since many long-term care organisations work with self-managing teams, challenges exist at team level. Therefore, there is a need to implement an intervention aimed at maintaining and improving the sustainable employability of staff on team level.

**Methods:**

We developed a participatory workplace intervention, the Healthy Working Approach. In this intervention teams will uncover what problems they face related to autonomy, relatedness and competence in their team, come up with solutions for those problems and evaluate the effects of these solutions. We will evaluate this intervention by means of a two-arm randomized controlled trial with a follow-up of one year. One arm includes the intervention group and one includes the waitlist control group, each consisting of about 100 participants. The primary outcome is need for recovery as proxy for sustainable employability. Intervention effects will be analysed by linear mixed model analyses. A process evaluation with key figures will provide insight into barriers and facilitators of the intervention implementation. The Ethical Committee Social Sciences of the Radboud University approved the study.

**Discussion:**

This study will provide insight in both the effectiveness, and the barriers/facilitators of the implementation process of the Healthy Working Approach. The approach is co-created with long-term care workers, focuses on team-specific challenges, and is rooted in the evidence-based participatory workplace approach and Self-Determination Theory. First results are expected in 2022.

**Trial registration:**

Netherlands Trial Register, NL9627. Registered 29 July 2021 - Retrospectively registered.

## Background

Sustainable employability of the workforce is a growing concern for many sectors, but especially for long-term care. On the one hand, the aging population requires more long-term care, whereas on the other hand the number of caregivers relative to older adults is declining. It appears difficult to attract new personnel while retaining current staff, especially for the direct care workers like nurses and personal care workers [1]. The direct care workforce currently working in long-term care experience several difficulties at work like shortage of staff, high physical and emotional demands, heavy workload, scheduling challenges, insufficient supervision and limited training and career advancement prospects which relate to job dissatisfaction and high turnover [2–7]. With many employees leaving their current profession, the burden increases for those who remain, which poses a threat to long-term care workers’ sustainable employability [1, 8–12].

Many scholars argue that in order to improve sustainable employability of current staff, the focus should be on improving working conditions and job quality [1, 13]. This is in line with needs expressed by long-term care workers for reducing job demands (e.g. reducing workload, diminishing rules and regulations) and improving job resources (e.g. more autonomy, appreciation and training possibilities) [14]. Having too many job demands and too little job resources to buffer against those demands, is known to have a negative impact on employee health and organisational outcomes [15]. Having too high job demands is not only energy depleting in itself, but also frustrates employees in the fulfilment of their basic psychological needs for autonomy, relatedness and competence [16]. According to the Self-Determination Theory precisely those three basic psychological needs are required for humans to actualize their potential [17]. *Autonomy* is experienced when people act from their own interests and values, and feel as if their behaviour is an expression of themselves [18]. *Relatedness* involves the feeling of being connected and belonging to others and to experience a sense of communion [18]. *Competence* refers to feeling effective in social interactions and experiencing opportunities to practice and express ones capabilities [18]. Satisfaction of these three basic needs relates to a variety of beneficial outcomes for employees, which ultimately benefit their sustainable employability [16, 19–21]. It is therefore important to foster the satisfaction of the needs for autonomy, relatedness and competence for employees to thrive at work [16].

In long-term care, many organisations have chosen an organisational structure that empowers its staff by working with self-managing or self-directing teams. Self-managing teams are autonomous teams, where the responsibility for providing good quality care and optimizing the wellbeing of the resident lies within the team instead of with a supervisor or team leader. The teams can decide on a range of tasks such as rostering, planning, individual and team performance monitoring, professional development and care delivery [1]. In self-managing teams different types of professionals (e.g., nurses, nursing assistants, social workers, therapists) work together to realise good quality of care for the residents. Long-term care teams in our paper therefore refer to a group of professionals responsible for accommodating care and assistance to a number of physically and/or cognitively impaired, typically older, people. As teams and residents vary, there is considerable variation in how self-managing teams organize the care for their residents [22]. Consequently, challenges with regards to the organisation of work likely differ between teams. Therefore, team level interventions are preferred over individual level interventions to obtain a sustainable long-term care workforce [23]. Moreover, given the diversity between teams within long-term care facilities, a one size fits all (teams) approach is not likely to be effective in protecting and stimulating sustainable employability [24, 25]. For the purpose of this study, we developed the Healthy Working Approach in close collaboration with long-term care workers. The Healthy Working Approach consists of a participatory workplace intervention at team level. The participatory approach is an established method aimed at promoting health and safety at work by means of a number of defined process steps, guided by a facilitator. The aim of these steps is to identify the most important bottlenecks at work and to come up with appropriate solutions using a concrete plan of action and equal input from all stakeholders [26]. This approach likely results in high degree of acceptance of proposed changes, which increases the likelihood that new way(s) of working implemented based on the intervention will be sustained over time [27, 28]. A participatory workplace intervention appears to be effective in improving several health issues, like hand eczema and back pain at the organisational level in various health settings [29–31]. In our Healthy Working Approach we used the Self-Determination Theory as the foundation for the focus of the participatory workplace intervention. The aim of our Healthy Working Approach is therefore to improve sustained employability of long-term care workers through improving fulfilment of their basic psychological needs at work. To gain insight into the effectiveness of the intervention, we will evaluate both the process of the Healthy Working Approach and the effects on sustainable employability in long-term care organisations working with self-managing teams. The primary outcome is need for recovery, also referred to as fatigue after work, as proxy for sustainable employability. Need for recovery is known to be a precursor for health problems that have a strong negative effect on the health and well-being of employees [32–35]. Moderate and high levels of burnout for example are highly prevalent in long-term care workers and are a long-term effect of short-term desires to be relieved from work demands in order to restore (also known as need for recovery) [36, 37]. Prolonged and increased need for recovery can therefore be seen as an early sign of a decreasing sustainable employability.

The main research question is:

What are the effects of the Healthy Working Approach on the sustainable employability of long-term care workers over a one year follow-up?

The main objectives of this study are:

- To gain insight into the effectiveness of the Healthy Working Approach on need for recovery in long-term care workers over one year;
- To gain insight into the process of implementing the Healthy Working Approach in long-term care teams.

## Methods

### Study Design

This is a randomised controlled trial with an intervention group and waitlist control group. There will be four measurement moments: at baseline (T0), 6 months (T6), 9 months (T9) and 12 months after baseline (T12). Data collection started in May 2021. The study protocol was approved by the Ethical Committee Social Sciences of the Radboud University (number: ECSW-2021-012).

### Setting

This study will be conducted in long-term care organisations that work with self-managing teams in the Netherlands.

### Procedure

Long-term care organisations are invited to participate in the study. After permission has been obtained, care teams are invited to participate via internal communication tools (e.g., intranet), and a personal e-mail with a link to the baseline questionnaire (T0). The questionnaire starts with an eligibility check followed by a digital informed consent. After participants have given their consent and their contact details, they are redirected to the main questionnaire. Information and questionnaires are also available in print. To enhance the response rate, the researcher will contact the teams to ask them how the research team can support them in filling out the questionnaires, for example by visiting with a laptop, or by bringing over hardcopy questionnaires. Recruitment of participants will continue until target sample size is reached.

### Participants

All professionals who both directly and indirectly contribute to providing good quality of care to residents in long-term can participate in the study. The eligibility check in the first questionnaire verifies whether the individual meets the following inclusion criteria:

- The long-term care worker is able to read and understand the Dutch language;
- The long-term care worker is minimally 18 years old.

Long-term care workers are excluded from participation when meeting the following exclusion criteria:

- The long-term care worker is on sick leave for one month or more before completing the baseline questionnaire;
- The employment contract of the long-term care worker ends within six months after completing the baseline questionnaire.

Because the intervention is at team level, teams are included in the study if at least a third of the team members have completed the baseline questionnaire and at least three team members are willing to represent their team in the three meetings of the intervention (i.e. take part in the working group).

### Participant involvement

Long-term care workers are involved in the design of the Healthy Working Approach by means of interviews in which their needs regarding sustainable employability are explored. Their needs formed the basis for the development of the Healthy Working Approach, which we presented to several teams/team members in order to check the feasibility and acceptability of the intervention. Key persons involved in healthy working within the long-term care organisation are involved in designing the recruitment process of facilitators and participating teams as well as in an appropriate dissemination plan for the facility. Outcomes are chosen based on interviews with employees and key figures within the long-term care organisation.

### Intervention: the Healthy Working Approach

The intervention consists per team of three meetings of one hour each led by a facilitator, who is a trained employee from within the long-term care organisation. Teams choose at least three representatives of their team to take part in a working group that will attend the meetings. The working group is responsible for representing the entire team and for reporting back to the team. The approach aims to result in improvements that benefit the whole team.

#### Meeting 1: Problem analysis (± one month after baseline)

In the first meeting, the working group starts with a brainstorm about problems within their team related to healthy working in the context of the three basic psychological needs, namely autonomy, relatedness and competence. Next, the working group starts prioritizing and chooses two or three problems that have great impact (high severity and frequency) for the entire team. The working group reports the chosen problems to the entire team, to make sure that everyone agrees these are problems that need to be tackled within their team. The facilitator ensures a safe and confidential environment, where everyone and every opinion is equal and respected.

#### Meeting 2: Solutions & action plan (one to two weeks after meeting 1)

After two or three problems that are agreed upon by the whole team, the working group brainstorms about solutions for these problems in the second meeting. The brainstorm about solutions starts broad and may include different sorts of solutions (technical or organisational solutions, working conditions or support). Potential solutions are prioritized based on criteria simplicity, feasibility, support, practicability and expected effectiveness. The working group formulates an implementation plan for the best one or two solutions for each problem. The plan includes specific, measurable, achievable, relevant and timebound (SMART) actions. The working group reports the solutions back to the entire team, to make sure that everyone knows which actions are agreed upon and what is expected from them.

#### Meeting 3: Implementation and evaluation (one to two months after meeting 2)

In the implementation phase, teams are guided and supported by the facilitator where necessary in carrying out the solutions. In the third meeting, the implementation status of the solutions are discussed (implemented, not implemented, in progress). If needed, solutions or additional steps will be discussed to improve the implementation status of solutions.

### Allocation of intervention and waitlist control group

Randomisation will take place at team level. The randomisation is performed by a research assistant who has no knowledge about the teams, using randomizer.org. In this tool, teams are inserted as pairs: of each pair one team is assigned to the intervention group and the other to the control group. The waitlist control group will start the intervention after completing the 12-month follow-up questionnaire. The intervention and control groups are aware of their own allocation status, but not of the allocation status of other teams. The allocation status of teams are known to the researchers involved in this study. In case of close collaboration between multiple teams within a department, the department is randomised to avoid contamination between these teams.

### Effectiveness evaluation

The Healthy Working Approach will be evaluated in a randomised controlled trial with one year of follow-up, including four measurement moments in which the following primary and secondary outcomes will be measured.

### Outcomes

#### Primary outcome

*Need for Recovery* will be measured with the 11 dichotomous items (0 *no* or 1 *yes*) of the Questionnaire on Psychosocial Job Demands and Job Stress [38]. The need for recovery score is a percentage score (0 to 100) of positive answers on the items. Higher scores indicate a higher degree of need for recovery after work. The scale has shown to possess good psychometric qualities in terms of (content) validity and internal consistency (Cronbach’s alpha ranging from .83 to .92) [39].

#### Secondary outcomes

Within the intervention teams will uncover bottlenecks regarding their need for autonomy, competence and relatedness. We expect most of the bottlenecks to focus on reducing job demands and improving job resources, which benefit the satisfaction of the three needs at work and ultimately lessen the need for recovery. Therefore, the satisfaction of the needs for autonomy, competence and relatedness, as well as several psychosocial job factors were measured as secondary outcomes.

*Satisfaction of the needs for Autonomy, Competence and Relatedness* will be measured with 16 items of the validated Work-related Basic Need Satisfaction Scale on a 5-point scale (ranging from 1 *totally disagree* to 5 *totally agree*) [40]. Mean scores will be calculated for the subscales autonomy (6 items), competence (4 items) and relatedness (6 items). The Work-related Basic Need Satisfaction Scale is widely used and validated in the Dutch language [40]. The scales for autonomy, competence and relatedness satisfaction show good reliabilities with Cronbach’s alpha’s of on average .81, .85 and .82 respectively [40].

*Work engagement* will be measured with 9 items of the Utrecht Work Engagement Scale on a 7-point scale (ranging from 0 *never* to 6 *always)* [41]. Mean scores will be calculated for the subscales vigour (3 items), absorption (3 items) and dedication (3 items), as well as an average total score work engagement (9 items). The Utrecht Work Engagement Scale has shown good internal consistency and test-retest reliability. Across different nations the Cronbach’s alpha of the scale is satisfying with a value of .86 [41].

*Physical demands* will be measured with 3 items of the Netherlands Working Conditions Survey on a 3-point scale (ranging from 1 *no*, 2 *yes, sometimes* and 3 *yes, regularly)* [42]*.* With monitoring data of over 15 years, the Netherlands Working Conditions Survey is a well-known and used tool to assess working situations of Dutch employees [42]. For this study a mean score will be calculated, whereby a higher score means more physical demands (more pushing/pulling, repetitive movements and uncomfortable working postures).

*Quantitative job demands* will be measured with 3 items of the Netherlands Working Conditions Survey on a 4-point scale (ranging from 1 *never* to 4 *always)* [42]*.* A mean score will be calculated, whereby a higher score means a higher workload (working more quickly, having a lot of work and working extra hard). Cronbach’s Alpha of the scale is good with a value of .80 [42].

*Self-reported health* will be measured with the Dutch translation of 2 items from the third version of the Copenhagen Psychosocial Questionnaire [43, 44]. One item askes participants to rate their health either as *excellent* (100), *very good* (75), *good* (50), *fair* (25) or *poor* (0). The second item askes them to give points to their present state of health (0 for *worst* and 10 for *best conceivable state of health*). A higher scores on each item reflects a better general health. The Copenhagen Psychosocial Questionnaire is internationally widely used to study work characteristics and is recently well validated in Dutch [44, 45].

Several psychosocial work factors will also be measured with items from the Dutch translation from the third version of the Copenhagen Psychosocial Questionnaire [43, 44]. It concerns the core items for *influence at work* (1 item), *job satisfaction* (1 item), *possibilities for development* (2 items) and the long measurement of *social support from colleagues* (3 items). Answer categories of all factors range from 0 *rarely* to 100 *always*. Mean scores will be calculated in case of multiple items per subscale, whereby a higher score means more of the psychosocial work factor at hand. Previous research shows the items and scales of this questionnaire are reliable [44, 45].

#### Prognostic factors

At baseline, several prognostic factors will be included in the survey, namely: age, gender, educational level, job title, years employed and type of contract (temporary or permanent), number of contractual working hours, hours of informal care provision per week in the last six months and frequency and total number of working days of sickness absence in the last six months.

### Participant time line

**Fig. 1:**
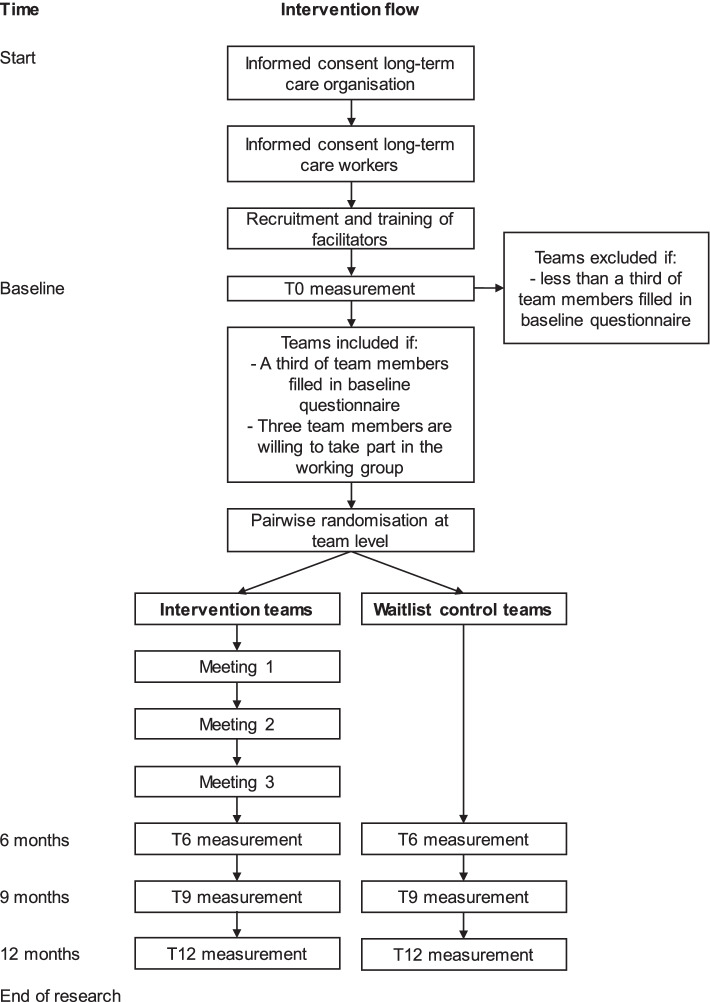
Timeline detailing the recruitment process, enrollment, randomization, and the different measurements and intervention meetings over time for participants of the Healthy Working Approach

Figure 1 shows an overview of the time line for participants in the intervention and waitlist control group. To promote participant retention and the completion of follow-up questionnaires, we will apply response-enhancing measures by offering teams that achieve a 75% response rate in the follow-up measurements a gift of their choice (e.g. fruit or flowers for the team).

### Sample size calculation

The primary outcome of this study is need for recovery [38], which we used for the calculation of the sample size. The mean need for recovery score of employees in occupational health services is 27.30 (SD = 29.75) on a scale of 0 to 100 [39]. The minimum relevant difference on the scale is 12 [46, 47]. An intraclass correlation (ICC) of 0.025 is assumed because previous studies have shown that ICCs at the workplace level for health-related outcomes are generally small [48–50]. Using the ICC for teams, a power of 80% and an alpha of 5%, the power analysis showed that a sample size of 161 employees will be needed to detect a difference of at least 12 points. Taking into account a 25% withdrawals and dropouts, the entire study population must consist of 202 long-term care workers (101 in intervention and 101 in control group).

### Data management and analysis

Before the start of the project, all issues of data management will be addressed in a data management plan. For this, Radboud University has a tool, that includes feedback from Research Data Management (RDM) Support. Training and support in writing a data management plan are offered by the section RDM Support and the data steward of the institute. In order to check whether the research has been carried out properly and reliably, authorised persons within the Behavioural Science Institute or Radboud University and (inter)national supervisory authorities (for example, the Netherlands Authority for the Protection of Personal Data) are able to inspect the data. While research is ongoing, data will be stored on the Radboud University’s network. The server space allows for managed access to and the sharing of data between and among partners and guests during the project. Safe and secure storage of data is guaranteed by the Information Technology security and safety protocols of the campus network.

We will perform descriptive analyses (means, standard deviation, frequencies) on all outcomes and covariates. For the main analysis, we will perform linear mixed model analyses with need for recovery as primary outcome, and group (intervention/control) x time interaction as independent variable, taking into account potential confounding prognostic factors. Potential confounders are included in the model when they account for at least 10% change in the main effect size of the group x time interaction. We will take into account nesting of the data. Similar analyses will be done with the secondary outcome measures.

Main analyses are performed according to the “intention to treat” principle and the unit of analysis is on the individual level. In addition, we will perform a per-protocol analysis to take into account teams in the intervention group that did not participate in the intervention or did not implement the intervention as planned. Any cases of missing data will be dealt with by imputation.

### Process evaluation

We will perform a process evaluation to evaluate the barriers and facilitators of the implementation process of the Healthy Working Approach using a combination of quantitative and qualitative methods. For the process evaluation, we will gain insight into recruitment, reach, dose received, dose delivered and fidelity in order to monitor the adherence to the procedures [51]. In addition, we will investigate barriers and facilitators of the implementation and satisfaction with the Healthy Working Approach. Table 1 provides an overview of who is involved in what element of the evaluation to provide an insight in the effectiveness, barriers and facilitators of the implementation process.

**Table 1:** Overview of process and evaluation set up of the Healthy Working Approach by means of source, type of info and data collection method

Source	Type of info	Data collection method
Facilitators	Evaluation of the training and coaching, and how they perceived their role during the working group meetings	Interviews
Working group members	Evaluation of the content and process of the three meetings	Evaluation forms and interviews
Intervention group participants	Evaluation of the Healthy Working Approach and how they experience the implementation of solutions	Additional questions during the 6 and 9 month follow-up questionnaire
Stakeholders in the long-term care organisation	Evaluation of the Healthy Working Approach	Interviews

## Discussion

This study addresses a compelling need for change in working conditions within long-term care to retain and improve the sustainable employability of its staff. Since many organisations work with self-managing teams, a team level approach is recommendable. Therefore, the Healthy Working Approach focuses on team-specific challenges, which likely increases the acceptability of implemented solutions. To our knowledge the Healthy Working Approach is one of the first participatory interventions at team level, cocreated with long-term care workers. The intervention has a strong basis, since it builds on the successful participatory workplace approach [29–31] and draws from the Self-Determination Theory which recognizes the importance of fostering the needs for autonomy, relatedness and competence for people to thrive at work [16]. By conducting both an effect- and a process evaluation, we will provide insight in both the effectiveness as well as the barriers/facilitators of the implementation process of the Healthy Working Approach.

One of the challenges in this design will be to include teams that perceive staff shortage and related challenges, as participating in the intervention requires time and focus. Even though these teams can specifically benefit from the intervention, these issues can prevent teams from participating. During recruitment we will emphasise that even though participating is a time investment, it is a way to tackle current issues (and therefore likely to be beneficial long-term).

Although we use a randomised controlled design, where randomisation takes place at the level of the department or team, contamination cannot be completely avoided. Communication within the organization and between teams during the study can cause waitlist control teams to get knowledgeable of the intervention and its implications. Because waitlist groups are informed about the study, give their consent, are allocated to the waitlist control group, and fill in questionnaires they are arguable not completely untreated [52]. To avoid most contamination and disclosure of information of individual participants we plan to disseminate the results on group level after the intervention within participating organisations, in peer-reviewed journals, and at academic conferences. First results from the study are expected in 2022.

## Data Availability

Data sharing is not applicable to this article as no datasets were generated or analysed during the current study. A de-identified dataset will be made available once the study is completed.

## Abbreviations

ICC: Intraclass Correlation
RDM: Research Data Management
SD: Standard Deviation
SMART: Specific, Measurable, Achievable, Relevant and Timebound
